# The Role of mTOR and eIF Signaling in Benign Endometrial Diseases

**DOI:** 10.3390/ijms23073416

**Published:** 2022-03-22

**Authors:** Tatiana S. Driva, Christoph Schatz, Monika Sobočan, Johannes Haybaeck

**Affiliations:** 1 Institute of Pathology, Neuropathology and Molecular Pathology, Medical University of Innsbruck, 6020 Innsbruck, Austria; tatiana.driva@student.i-med.ac.at (T.S.D.); christoph.schatz@i-med.ac.at (C.S.); 2 Division of Gynecology and Perinatology, University Medical Centre Maribor, 2000 Maribor, Slovenia; monika.sobocan@gmail.com; 3 Department of Obstetrics and Gynecology, Faculty of Medicine, University of Maribor, 2000 Maribor, Slovenia; 4 Wolfson Institute of Preventative Medicine, Barts CRUK Cancer Centre, Queen Mary University of London, Charterhouse Square, London EC1M 6BQ, UK; 5 Diagnostic and Research Center for Molecular Biomedicine, Institute of Pathology, Medical University of Graz, 8010 Graz, Austria

**Keywords:** mTOR signaling, eIFs, adenomyosis, endometriosis, endometritis, typical endometrial hyperplasia

## Abstract

Adenomyosis, endometriosis, endometritis, and typical endometrial hyperplasia are common non-cancerous diseases of the endometrium that afflict many women with life-impacting consequences. The mammalian target of the rapamycin (mTOR) pathway interacts with estrogen signaling and is known to be dysregulated in endometrial cancer. Based on this knowledge, we attempt to investigate the role of mTOR signaling in benign endometrial diseases while focusing on how the interplay between mTOR and eukaryotic translation initiation factors (eIFs) affects their development. In fact, mTOR overactivity is apparent in adenomyosis, endometriosis, and typical endometrial hyperplasia, where it promotes endometrial cell proliferation and invasiveness. Recent data show aberrant expression of various components of the mTOR pathway in both eutopic and ectopic endometrium of patients with adenomyosis or endometriosis and in hyperplastic endometrium as well. Moreover, studies on endometritis show that derangement of mTOR signaling is linked to the establishment of endometrial dysfunction caused by chronic inflammation. This review shows that inhibition of the mTOR pathway has a promising therapeutic effect in benign endometrial conditions, concluding that mTOR signaling dysregulation plays a critical part in their pathogenesis.

## 1. Introduction

Adenomyosis, endometriosis, endometritis, and typical endometrial hyperplasia are all non-cancerous conditions that originate from the endometrium and affect a substantial part of the female population. They are often associated with endocrine derangement and develop upon high levels of estrogen or estrogen dominance (estrogen unopposed by progesterone) [1,2,3]. Although these disorders are considered benign, they have malignant potential and may increase the risk of developing ovarian and uterine tumors [4,5,6]. Importantly, all four of these endometrial diseases are common causes of severe pelvic pain, menstrual disorders, and female infertility. Due to this, they have a serious impact on women’s quality of life [7,8,9,10].

The molecular mechanisms that underlie the pathogenesis of benign endometrial diseases have not yet been fully elucidated. Current knowledge about their biological background brings the role of the mammalian target of the rapamycin (mTOR) signaling pathway to the fore. In fact, aside from its well-known importance in malignant endometrial disorders [11,12,13], mTOR signaling and its associated eukaryotic translation initiation factors (eIFs) seem to play a critical role in the development of non-cancerous endometrial diseases as well. The purpose of this review is to illuminate the involvement of mTOR signaling in the pathogenesis of adenomyosis, endometriosis, endometritis, and typical endometrial hyperplasia and to address its potential as a therapeutic target for these conditions.

### 1.1. Overview of mTOR Signaling and Its Correlated eIFs

The mammalian target of rapamycin (mTOR) is a human serine/threonine protein kinase with multiple biological functions that determine cell growth, metabolism, proliferation, and survival [14]. As implied by its name, mTOR is the functional target of rapamycin, a drug firstly described as a potent antifungal metabolite that was later proven to possess immunosuppressive and antitumorigenic properties in humans [15]. The mTOR forms two structurally and functionally distinct multi-molecular complexes called the mammalian target of rapamycin complex 1 (mTORC1) and mammalian target of rapamycin complex 2 (mTORC2). mTORC1 is composed of mTOR, Raptor, mLST8/GβL, PRAS40, and DEPTOR, while mTORC2 consists of mTOR, Rictor, mLST8/GβL, PRR5, DEPTOR, and SIN1 [16].

mTORC1 is the most well-characterized mTOR complex and the one that is sensitive to rapamycin treatment [16,17]. Its function can be stimulated by various environmental signals such as growth factors, nutrients, oxygen deprivation, and energy supply [18]. The most important regulation of mTORC1 is mediated by growth factors such as insulin via the PI3K/AKT pathway [19]. Briefly, the binding of growth factors to their receptors in the cell membrane activates the phosphatidylinositol-3-kinase (PI3K), which in turn converts phosphatidylinositol-4,5-phosphate to phosphatidylinositol-3,4,5-phosphate (PIP3), a process antagonized by the tumor suppressor protein phosphatase and tensin homolog (PTEN) [20]. PIP3 then activates protein kinase B (AKT), which inhibits the complex formed by the tuberous sclerosis complex proteins 1 and 2 (TSC1-TSC2), thereby promoting mTORC1 activation [21]. Apart from PI3K/AKT pathway, another important effector upstream of mTORC1 is adenosine monophosphate (AMP)-activated protein kinase (AMPK), a kinase sensitive to changes in cellular energy levels. AMPK acts by suppressing mTORC1 signaling either by TSC1-TSC2 stimulation or via direct inhibition of Raptor [18].

After being activated, mTORC1 coordinates protein synthesis through p70S6 Kinase 1 (S6K1) and eIF4E Binding Protein 1 (4EBP1) phosphorylation. Phosphorylated 4EBP1 then dissociates from the eukaryotic translation initiation factor 4E (eIF4E), allowing the latter to participate in the formation of the eIF4F complex and thus in the translation initiation [22]. On the other hand, phosphorylated S6K1 also promotes translation initiation via eIF4B activation and additionally regulates translation elongation by modulating the function of eEF2K [23]. Apart from cell growth and proliferation induced by 4EBP1 and S6K1, respectively, mTORC1 is also well-known as an autophagy inhibitor. In a nutrient-rich environment, mTORC1 suppresses the process of autophagy via inactivation of the factors ULK1/2 and ATG13 [24].

Apart from eIF4E and eIF4B, mTORC1 is involved in translation initiation through other eukaryotic translation initiation factors (eIFs), such as eIF2a and eIF3e. In addition to the canonical pathway previously described, mTORC1 can also regulate autophagy in a non-canonical manner by interacting with eIF2a signaling. More precisely, inhibition of mTORC1 activates the phosphatase PP6C [25], which in turn forms a complex with GCN2 that stimulates eIF2a signaling and, therefore, autophagy [26]. Furthermore, mTORC1 achieves S6K1 phosphorylation after recruitment to eIF3 [27], from which S6K1 then dissociates in order to phosphorylate its substrates. Interestingly, suppression of eIF3e seems to enhance S6K1 activity and thereby mTORC1 signaling [28].

In contrast to mTORC1, the function and regulation of mTORC2 have only been minimally characterized. mTORC2’s most important function is AKT phosphorylation, while it can also activate several members of the AGC family, and thereby control the organization of the cytoskeleton and cell migration [29,30]. Unlike mTORC1, mTORC2 activity is enhanced by TSC1–TSC2 complex [21]. Additionally, mTORC2 is not sensitive to acute treatment with rapamycin, but it can be inhibited after long-term treatment [31].

### 1.2. Cross-Talk between Estrogen and mTOR Signaling Pathway

The PI3K/AKT/mTOR pathway is involved in estrogen signal transduction, thus contributing to the adverse effects of abnormal estrogen levels on endometrial homeostasis [32,33]. In fact, estrogen receptors (ERs) induce the expression of several upstream regulators of the PI3K/AKT/mTOR pathway, such as receptor ligands, receptor tyrosine kinases (RTKs), and signaling adaptors [34]. Furthermore, ERs can stimulate the PI3K/AKT/mTOR pathway not only by acting as transcription factors but also by physically interacting with effectors that are involved in the cascade. For example, estrogen bound ERα directly binds to the p85α regulatory subunit of PI3K and promotes its phosphorylation [35]. In addition, activated ERα attaches to Raptor, causing the translocation of the latter to the nucleus [36]. Other than that, estrogen induces PI3K signaling via stimulation of insulin-like growth factor 1 receptor (IGF-1R) [37]. In reverse, the mTOR pathway can also promote ER signal transduction. Specifically, mTORC1 leads to ERα phosphorylation and activation via S6K1 [36,38].

**Figure 1:**
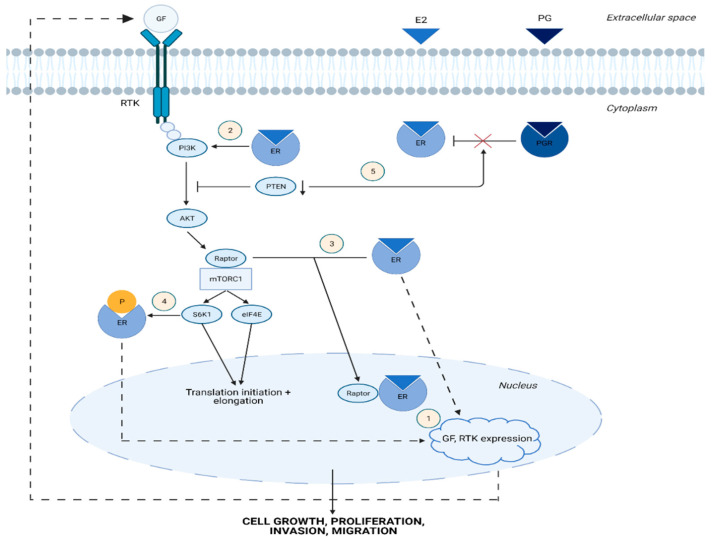
**Cross-talk between estrogen and PI3K/AKT/mTOR signaling pathway** (1) ERs induce the expression of upstream regulators of the PI3K/AKT/mTOR pathway. (2) Activated ER directly binds to PI3K and promotes its phosphorylation. (3) Estrogen-bound ER attaches to Raptor, causing its translocation to the nucleus. (4) mTORC1 leads to ER phosphorylation and activation via S6K1. (5) Low PTEN expression and thus aberrant PI3K/AKT/mTOR pathway activation may lead to progesterone resistance in the endometrium. E2, estradiol; PG, progesterone; ER, estrogen receptor; PGR, progesterone receptor; GF, growth factor; RTK, receptor tyrosine kinase; PI3K, phosphatidylinositol-3-kinase; PTEN, phosphatase and tensin homolog; AKT, protein kinase B; Raptor, regulatory-associated protein of mTOR; mTORC1, mammalian target of rapamycin complex 1; S6K1, p70S6 Kinase 1, eIF4E, eukaryotic translation initiation factor 4E. Graphic created with BioRender.com.

Estrogen signaling plays a critical role in the pathogenesis of endometrial diseases, as it enhances the potential for proliferation, invasion, and migration of endometrial cells. [32,35]. These characteristics are not only important for the development of malignant endometrial disorders but are also requisite for benign conditions, such as adenomyosis, endometriosis and typical endometrial hyperplasia [39]. The mTOR pathway is closely linked to estrogen signaling and may therefore play an important role in the derangement of endometrial function. Thus, it is worth clarifying the association of this signaling cascade with the main benign endometrial diseases (Figure 1(1–4)).

### 1.3. Brief Outline of mTOR Pathway Dysregulation in Malignant Endometrial Diseases

The role of mTOR signaling in endometrial carcinogenesis has been extensively investigated in recent decades. PI3K/AKT/mTOR pathway derangement occurs in 80–95% of Type I or endometrioid endometrial carcinomas [40], with PTEN being the most commonly mutated gene in this type of cancer [41]. Interestingly, PIK3CA is frequently commutated with PTEN, implying a synergic effect of both genes on AKT activation during endometrial tumorigenesis [42]. Besides Type I, PI3K/AKT/mTOR pathway dysregulation also contributes to the development of Type II endometrial carcinoma, as the pathway has been shown to interact with p53 signaling in uterine serous carcinoma cells. Furthermore, alterations in PI3K and RTKs are often detected [43], while mTORC2 upregulation and loss of TSC2 have also been observed in endometrial carcinoma cells [44,45]. Apart from endometrial carcinomas, aberrations in mTOR signaling have also been noted in endometrial stromal sarcomas (ESSs). Recent studies [46,47] have reported upregulation of PIK3-AKT signaling and alterations of the mTOR-NF2-AKT pathway in ESS, while inhibition of PI3K and mTOR has been shown to restrain growth in an ESS cell line [48]. The prominent role of the PI3K/AKT/mTOR pathway in endometrial tumorigenesis makes it an ideal therapeutic target for this type of cancer. Inhibitors of this signaling cascade have shown promising results in preclinical models of endometrial cancer and multiple clinical trials examining their efficacy in endometrial cancer patients have been and continue to be conducted [11,40,42]. Current research data have shed light on the importance of mTOR signaling for the development of non-cancerous endometrial diseases as well, suggesting it as an effective therapeutic target for these conditions.

## 2. mTOR and eIF Signaling in Benign Endometrial Diseases

### 2.1. mTOR Signaling in Adenomyosis

Adenomyosis refers to the uterine disease in which endometrial tissue invades the myometrium. It is considered an estrogen-dependent condition, as it is characterized by high estrogen levels that contribute to its pathogenesis [1,32,49]. Estrogen acts by stimulating the PI3K/AKT/mTOR pathway (Figure 1) [32,35,50], which is known to be activated in adenomyosis, leading to endometrial epithelial cell invasion and migration [51].

Multiple studies [51,52,53,54,55,56,57] have reported the involvement of upstream regulators of the mTOR pathway in the development of adenomyosis. Protein deglycase DJ-1, a protein associated with cancer and Parkinson’s disease, is increased in adenomyotic lesions, where it controls cell proliferation, migration, and angiogenesis by inducing AKT phosphorylation [52]. Furthermore, mTOR signaling is dysregulated in adenomyosis due to the altered expression of non-coding RNAs. The term “non-coding RNAs” (ncRNAs) covers a plethora of RNA molecules that do not code for proteins [58]. In adenomyosis, the ncRNAs known to influence the disease progression via modulating the PI3K/AKT/mTOR pathway are microRNAs (miRNAs) and long non-coding RNAs (lncRNAs) [51,53,54]. Briefly, miRNAs are short RNA molecules that regulate gene expression by binding to messenger RNAs (mRNAs) and hampering their function [59]. LncRNAs, on the other hand, are RNAs of more than 200 nucleotides with a multifunctional role in cell signaling, as they are involved in various processes such as gene regulation, translation, and RNA splicing [60]. The involvement of ncRNAs in the development and progression of adenomyosis is depicted in Table 1.

Besides upstream effectors, downstream regulators of mTOR signaling seem to play a significant role in adenomyosis as well. Vascular endothelial growth factor (VEGF) and hypoxia-inducible factor-1α (HIF-1α) are highly expressed in the ectopic endometrium of patients with adenomyosis, where they promote angiogenesis, thereby contributing to the progression of the disease [55]. Both VEGF and HIF-1α are upregulated by mTORC1 [52,64]. Specifically, mTORC1 induces HIF-1α synthesis through its substrates S6K1 and STAT3 and promotes the translation of both HIF-1α and VEGF via upregulation of eIF4E. Likewise, eIF4E mediates the expression of C/EBPβ [65,66,67], a transcription factor that induces endometrial stromal proliferation and differentiation in adenomyotic lesions [56,57].

mTOR signaling may also be involved in the pathogenesis of adenomyosis by promoting epithelial to mesenchymal transition (EMT) of endometrial epithelial cells. EMT is thought to play a crucial role in the establishment of the disease, as it enhances the invasiveness of endometrial cells, thus contributing to their translocation into the myometrium [1,68]. More specifically, TGF-β is increased in adenomyotic lesions and leads to EMT [1], possibly by activating mTORC2, one of its main downstream targets in this process [69]. In addition, the PI3K/AKT/mTOR pathway stimulates TWIST, a transcription factor that promotes the EMT phenotype in endometrial cells [12]. Finally, the protein kinase FAK may mediate EMT in adenomyosis via activation of the PI3K/AKT signaling cascade [70].

It follows that targeting the PI3K/AKT/mTOR pathway may be effective for adenomyosis treatment. Interestingly, Xue et al. observed that metformin could restrain stromal cell proliferation of adenomyotic lesions by downregulating the PI3K/AKT pathway through AMPK activation [32].

### 2.2. mTOR-Associated eIFs in Adenomyosis

Aberrant mTOR pathway activation is also reflected in the abnormal expression of mTOR-associated eIFs in adenomyosis. Gene expression and pathway analysis applied on eutopic endometrium from adenomyosis patients revealed significant dysregulation of eIF2 as well as eIF4 signaling in comparison to normal endometrium [71]. Specifically, eIF4a2, eIF3K, and eIF4b were expressed differently between adenomyotic and normal endometrium. Furthermore, Cai et al. noted a decreased expression of eIF3e in ectopic endometrium and suggested that this reduction may promote EMT via TGF-β1 activation, as they observed a negative correlation between eIF3 and TGF-β1, Snail, vimentin, and PCNA levels [72].

### 2.3. mTOR Signaling in Endometriosis

Endometriosis is an estrogen-dependent disorder in which endometrial tissue develops outside the uterine cavity, most commonly in the pelvic peritoneum, ovaries, and the pouch of Douglas [73]. The association between the PI3K/AKT/mTOR pathway and the pathogenesis of endometriosis has been proposed by multiple early studies [74,75,76,77,78]. Higher levels of pAkt have been detected in both eutopic and ectopic endometrial tissue of women with endometriosis compared to normal endometrium [75]. Previous research demonstrated increased transcription of the AKT1 and 4EBP1 genes in the eutopic endometrium of endometriosis patients, suggesting a possible role for these genes in endometrial growth outside the uterus [76]. Moreover, elevated expression of the mTOR activators AXL and SHC1 [78], as well as loss of PTEN, have been earlier described in endometriosis [77].

In recent years, the investigation of mTOR signaling in endometriosis is still evolving. Madanes et al. noted elevated PI3K expression and AKT phosphorylation as well as reduced PTEN levels in both ectopic and eutopic endometrium of endometriosis patients compared to normal endometrium. Interestingly, these findings were observed mainly in women with a minimal-mild stage of the disease, suggesting that the PI3K/AKT/mTOR pathway plays a critical role in the onset of endometriosis [79]. Similar conclusions were drawn from mouse models after detecting an increased formation of endometriosis lesions in the peritoneal cavity of mice carrying a PTEN deletion in PR-positive cells [80].

According to other studies [39,81], ectopic endometrial tissue exhibits persistent mTOR activity despite changes in estrogen and progesterone levels. In fact, the PI3K/AKT/mTOR pathway seems to regulate the response of ectopic endometrial tissue to progesterone and is probably associated with progesterone resistance, which is common in endometriosis [82,83]. In particular, Li et al. noted that suppression of PTEN expression by the microRNA miR-92a (see Table 1) and thus aberrant PI3K/AKT/mTOR pathway activation can lead to progesterone resistant endometriosis [61] (Figure 1(5)). Interestingly, mTOR signaling seems to be regulated by several microRNAs in endometriosis, and their function is presented in Table 1.

The frequent activation of the PI3K/AKT/mTOR pathway in endometriosis makes it an attractive therapeutic target in this disease. Therefore, scientists have attempted to evaluate the effects of inhibiting this pathway in restraining endometriosis. Leconte et al. noted a significant and dose-dependent decrease in the proliferation rate of deep infiltrating endometriotic stromal cells in both cell culture and mouse model experiments after treatment with the mTOR-inhibitor temsirolimus [84]. In another study, dienogest, a progestin medication approved for the treatment of endometriosis, was found to promote autophagy and apoptosis by suppressing AKT and ERK1/2 activity in ectopic endometrial cells [85]. Ren et al. indicated the repressive effects of rapamycin, the well-known mTOR inhibitor, on angiogenesis in endometriotic lesions. Specifically, they noted a significant reduction in VEGF expression and microvessel density in ectopic tissues of mice with peritoneal endometriosis after 2 weeks of rapamycin treatment [86]. Similarly, Cao et al. suggested that the vasodepressor ginsenoside Rg3 may limit the rate of ectopic endometrial tissue growth via suppressing the VEGF-mediated activation of the mTOR pathway [87].

Other reports have emphasized the role of mTOR signaling in endometriosis-associated carcinogenesis [88]. Cancer develops from endometriotic tissue, usually in the ovaries, involving either clear cell or endometrioid carcinoma histotypes [89]. In those histotypes, the tumor suppressor gene ARID1A that encodes a protein involved in chromatin remodeling is often inactivated. Interestingly, the loss of ARID1A is linked to the PI3K/AKT pathway dysregulation, and several studies suggest that activation of the PI3K/AKT/mTOR pathway in combination with ARID1A mutation are key events in the malignant transformation of endometriotic lesions [89,90,91]. Furthermore, Broadway et al. observed that specific components of the mTOR complexes, such as DEPTOR, are expressed in a common pattern among endometriosis and ovarian carcinoma tissues, again supporting the connective role of mTOR signaling between tumorigenesis and endometriosis [92].

### 2.4. mTOR-Associated eIFs in Endometriosis

mTOR pathway-related eIFs exhibit differential expression between normal and ectopic endometrium. Specifically, Cai et al. observed that eIF3e is underexpressed in ovarian endometriosis tissue, thus promoting EMT through the preferential translation of Snail, as they noted a negative correlation between eIF3 and TGF-β1, Snail, vimentin, and PCNA levels [93]. In addition, eIFs can serve as treatment targets for endometriosis. Choi et al. suggested that the PERK/eIF2α pathway is involved in the therapeutic mechanism of dienogest in the disease [94]. According to their study, dienogest increases the expression of CHOP, an apoptosis-mediator protein, via activation of eIF2α signaling. Considering that CHOP is known to induce cell apoptosis by repressing the mTOR pathway [95], it is possible that dienogest acts by inhibiting mTOR signaling through an eIF2α-mediated CHOP activation. Moreover, the flavonoids naringenin and chrysin exhibit a suppressive role in endometriotic cell lines by enhancing endoplasmic reticulum stress via eIF2α activation and PI3K/AKT pathway inhibition [96,97].

### 2.5. mTOR Signaling in Endometritis

Endometritis can be classified as acute or chronic, depending on the predominance of a neutrophilic or a lymphoplasmacytic infiltrate, respectively. Chronic endometritis (CE) often leads to endometrial dysfunction and therefore increases the risk of infertility and embryo implantation failure. Wang et al. demonstrated through a case-control study including women with CE and recurrent implantation failure (RIF) that mTORC1 is significantly under-expressed in inflamed endometrium [98]. It has also been suggested that mTOR pathway downregulation and subsequent increase in autophagy reduces endometrial receptivity and can be the root of the unsuccessful implantation that is observed in CE patients [99]. However, the role of the mTOR pathway in endometrial inflammation has not been fully elucidated. Contrary to the above-mentioned data, a double-blind, phase II randomized clinical trial revealed the beneficial effect of Sirolimus treatment on RIF patients with elevated Th17/Treg cell ratio [100]. Considering that CE leads to the predominance of Th17 over Treg immunity [98], the latter study suggests that mTOR pathway inhibition could potentially have an immunosuppressive impact on inflamed endometrium.

### 2.6. mTOR Signaling in Typical Endometrial Hyperplasia

Endometrial hyperplasia (EH) is an irregular proliferation of the endometrium that comprises alterations in the glandular architecture and endometrial gland-to-stroma ratio. According to the simplified World Health Organization (WHO) classification, EH can be categorized as hyperplasia with or without atypia, depending on whether cytologic atypia is present or not, respectively [101]. For the purpose of this review, only EH without atypia, which is presumed benign, will be analyzed.

EH arises from endometrial exposure to excessive levels of estrogen that are not opposed by progesterone, as can be seen in various conditions such as post-menopause, obesity, and polycystic ovary syndrome (PCOS) [3,101]. Considering that estrogen promotes DNA and protein synthesis in endometrial cells through mTOR signaling, EH could probably be associated with aberrant mTOR pathway activation [102]. Interestingly, the latter hypothesis has been suggested by multiple recent studies.

Bajwa et al. indicated the importance of mTOR signaling in advanced age-related EH. They observed an increased pS6 expression in hyperplastic endometrium collected from post-menopausal women compared to normal post-menopausal endometrium and noted a similar expression pattern of pS6 between hyperplastic and normal uterine tissue collected from aged mice [103]. Interestingly, treatment of aged mice with rapamycin led to a significant decrease in EH features. The association between EH and mTOR was strengthened in the same study by reporting the establishment of hyperplastic epithelium and abnormal glandular architecture in the uteri of mouse models carrying a PTEN deletion. Besides post-menopausal EH, the mTOR pathway also contributes to the pathogenesis of obesity-induced EH. Studying the uteri of obese mice, Sahoo et al. revealed elevated phosphorylation of VEGFR2 at Tyr1175 and increased pS6 expression in the hyperplastic endometrium. This observation was interpreted as upregulation of the mTOR pathway by VEGF since it is known that VEGF activates mTOR signaling via the VEGFR2 receptor [104].

**Figure 2:**
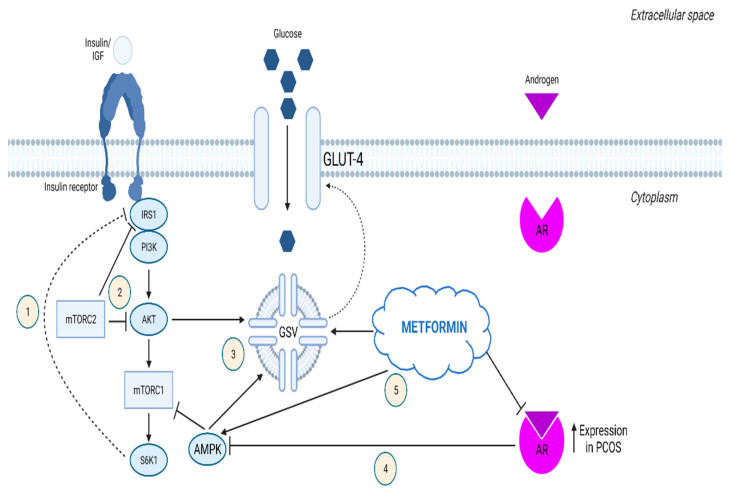
**mTOR signaling in PCOS-induced endometrial hyperplasia** Glucose metabolism in the uterus is of essential importance for normal endometrial function. Hyperinsulinemia and insulin resistance are major disorders involved in the pathogenesis of PCOS and are believed to contribute to the development of EH in women with this condition. (1) Normally, S6K1 phosphorylates IRS1, thereby preventing the association of IRS1 with the insulin receptor. This negative feedback mechanism desensitizes and protects the cell from further insulin stimulation. When mTORC1 is chronically activated, for example, due to excessive glucose consumption, the sustained downstream stimulation of S6K1 increases IRS1 phosphorylation. This decreases its responsiveness to insulin, thus rendering the cell insulin resistant [109]. (2) mTORC2 influences insulin sensitivity by impairing IRS1 and AKT function. (3) GLUT-4 expression and translocation to the plasma membrane are enhanced by AMPK and AKT, respectively [109,117]. (4) High androgen levels in PCOS restrain apoptosis and increase the proliferative potential of endometrial cells by stimulating mTOR signaling via AMPK inactivation. (5) Metformin restrains EH by suppressing the mTOR pathway through AMPK stimulation. It has also been shown that metformin increases GLUT-4 and decreases AR levels in the endometrium, probably via the mTOR pathway [118]. IGF, insulin-like growth factor; IRS1, insulin receptor substrate 1; PI3K, phosphatidylinositol-3-kinase; AKT, protein kinase B; mTORC2, mammalian target of rapamycin complex 2; mTORC1, mammalian target of rapamycin complex 1; S6K1, p70S6 kinase 1; AMPK, adenosine monophosphate (AMP)-activated protein kinase; GLUT-4, glucose transporter type 4; GSV, GLUT-4 storage vesicle; AR, androgen receptor3. Graphic created with BioRender.com.

mTOR signaling derangement also seems to have a pivotal role in the development of EH induced by PCOS. PCOS is a disorder characterized by chronic anovulation and aberrant androgen biosynthesis, conditions that both increase unopposed steroid levels and can therefore promote EH [105]. The mTOR pathway plays an important role in multiple pathways that are dysregulated in PCOS, including androgen action, insulin axis and cell apoptosis. High androgen levels in PCOS restrain apoptosis and increase the proliferative potential of endometrial cells by stimulating mTOR signaling via AMPK inactivation [106] (Figure 2(4)). Moreover, increased AKT phosphorylation has been observed in endometrial tissue coming from PCOS patients [74]. Another study has demonstrated that EH in patients with PCOS results from abnormal autophagy mechanisms [107], implying another possible link between mTOR and PCOS-induced EH since mTOR is a major autophagy regulator.

Glucose metabolism in the uterus is of essential importance for normal endometrial function. Hyperinsulinemia and insulin resistance are major disorders involved in the pathogenesis of PCOS and are believed to contribute to the development of EH in women with this condition [106,108]. Insulin resistance is known to derive from abnormal function of the mTOR pathway and in particular from overactive mTORC1 and aberrant mTORC2 signaling [105,109] (Figure 2(1,2)). Furthermore, Li et al. reported impaired glucose intake in hyperplastic PCOS endometrium, as they observed lower GLUT-4 expression in comparison to normal endometrium [106]. According to their study, this dysregulation of glucose metabolism results from overactivity of the insulin receptor/PI3K/Akt/mTOR signaling cascade (Figure 2(3)). Specifically, they noted an increase in GLUT-4 levels in combination with reduced levels of the main mTOR pathway components after treating EH tissues with metformin.

It has been well established that metformin restrains EH via mTOR pathway modulation [110], as it activates AMPK, a significant negative regulator of mTOR signaling [111]. In PCOS, metformin can increase GLUT-4 and reduce androgen receptor (AR) levels in the endometrium, probably through the mTOR pathway [106,112]. In addition, compared to normal rat endometrium, uteri of rat models of PCOS exhibit high mTORC1 accumulation, which can be restored to normal levels via metformin treatment [113] (Figure 2(5)). As reported by another study, metformin attenuates estrogen and tamoxifen induced EH by inhibiting S6K1 activation by mTOR [114]. Data derived from in vivo studies as well as clinical trials show that metformin is comparable to progestins for the treatment of simple endometrial hyperplasia [115,116]. The fact that metformin acts in the endometrium via downregulating the mTOR pathway highlights the importance of this signaling cascade in EH.

Apart from exposure to unopposed estrogen, EH can also result from treatment with tamoxifen, a selective estrogen receptor modulator (SERM) used for breast cancer therapy. Upregulation of mTOR signaling might be part of the mechanism by which tamoxifen induces abnormal endometrial growth [3]. Sequestosome1/p62 (SQSTM1/p62) is an autophagy-related adaptor protein and its expression is enhanced by mTORC1 [119]. Interestingly, a study showed higher expression of SQSTM1 in tamoxifen induced EH, in comparison to hyperplastic endometrium derived from women who had not received tamoxifen. In addition, the same study demonstrated a significant retreat of tamoxifen induced EH after inhibition of Nrf2, the upstream activator of SQSTM1 [120].

### 2.7. mTOR-Associated eIFs in Typical Endometrial Hyperplasia

It is noteworthy that eIF2α signaling, which is in close interaction with the mTOR pathway, seems to be involved in the pathogenesis of PCOS-induced endometrial hyperplasia. In fact, endoplasmic reticulum stress (ER) and consequent eIF2α pathway activation directly contribute to the pathogenesis of PCOS by affecting the total health of ovarian follicles and oocytes in several ways, such as induction of interstitial fibrosis, follicular atresia, and accumulation of advanced glycation end products (AGEs) in ovarian granulosa cells [121,122]. Furthermore, the eIF2α pathway is involved in insulin resistance, which enhances the development of EH in PCOS patients, as previously mentioned. In PCOS, high androgen levels may lead to impaired insulin secretion via promoting ER stress in β-cells and activating the eIF2α signaling cascade. Furthermore, under excess androgen conditions, eIF2α stimulates CHOP expression in insulin-secreting cells, thereby suppressing mTOR signaling and leading to apoptosis [123].

## 3. Conclusions

The importance of the PI3K/AKT/mTOR signaling cascade in endometrial carcinogenesis has been well established and research has proceeded on evaluating the efficacy of inhibiting this pathway for the treatment of endometrial malignancies. Based on this knowledge, we aimed to investigate the current data about the involvement of the mTOR pathway in the development of benign endometrial conditions. From this review, it follows that mTOR and eIF signaling are deranged in adenomyosis, endometriosis, endometritis, and typical endometrial hyperplasia and may serve as druggable targets in these life-impacting diseases. However, further studies are still needed to determine the full spectrum of mTOR function in non-cancerous endometrial conditions.

## Figures and Tables

**Table 1:** Overview of non-coding RNA molecules identified interacting with upstream regulators of the mTOR pathway.

Endometrial Disease	Non-Coding RNA	Component	Impact on Signaling	Impact on Disease Progression
Adenomyosis	Linc-ROR [53]	PTEN downregulation	PI3K/AKT pathway upregulation	promotion of endometrial cell proliferation
	miR-17 [54]	3′UTR of PTEN	possible PI3K/AKT pathwayupregulation	promotion of endometrial cell apoptosis
	miR-10b [51]	3′UTR of PIK3CA	PI3K/AKT pathway downregulation	inhibition of endometrial cell invasion and migration
Endometriosis	miR-92a [61]	3′UTR of PTEN	possible PI3K/AKT pathway upregulation	promotion of progesterone resistance in endometriosis
	miR-194-5p [62]	3′UTR of STAT1	STAT1/mTOR pathway downregulation	inhibition of endometrial cell proliferation and invasion
	miR-106a-5p [63]	3′UTR of FOXC1	PI3K/AKT/mTOR pathway downregulation	inhibition of ectopic endometrial stromal cell proliferation, migration, and invasion

mTOR, mammalian target of rapamycin; PTEN, phosphatase and tensin homolog; PI3K, phosphatidylinositol-3-kinase; 3′UTR, 3′ untranslated region; PIK3CA, phosphatidylinositol-4,5-bisphosphate 3-kinase catalytic subunit alpha; STAT1, signal transducer and activator of transcription 1; FOXC1, forkhead box protein C1.

## Data Availability

Not applicable.
